# Supplementary material for: Nutritional Biomarkers for the Prediction of Response to Anti-TNF-α Therapy in Crohn’s Disease: New Tools for New Approaches
Source: Nutrients. 2024 Jan 17;16(2):280. doi: 10.3390/nu16020280 (PMC10818399; doi:10.3390/nu16020280)
Supplement: Supplementary file 1 [file nutrients-16-00280-s001.zip › Supplementary File S1 questionnaire.pdf]

nutrients MDPI

Supplementary Material Table S1

Food frequency questionnaire

Name & surname: ____________________ ID subject: ________________

Date: ____________________________ Day of the week: __________________

DAY 1

Before breakfast:

TIMES	PLACE	DESCRIPTION OF BEVERAGES / FOODS AND TYPE OF PREPARATION	QUANTITY

Breakfast:

TIMES	PLACE	DESCRIPTION OF BEVERAGES / FOODS AND TYPE OF PREPARATION	QUANTITY

Morning Snack:

TIMES	PLACE	DESCRIPTION OF BEVERAGES / FOODS AND TYPE OF PREPARATION	QUANTITY

Lunch:

TIMES	PLACE	DESCRIPTION OF BEVERAGES / FOODS AND TYPE OF PREPARATION	QUANTITY

Afternoon snack:

TIMES	PLACE	DESCRIPTION OF BEVERAGES / FOODS AND TYPE OF PREPARATION	QUANTITY

Dinner:

TIMES	PLACE	DESCRIPTION OF BEVERAGES / FOODS AND TYPE OF PREPARATION	QUANTITY

After dinner:

TIMES	PLACE	DESCRIPTION OF BEVERAGES / FOODS AND TYPE OF PREPARATION	QUANTITY

During the night:

TIMES	PLACE	DESCRIPTION OF BEVERAGES / FOODS AND TYPE OF PREPARATION	QUANTITY

Comments (e.g. recipes)

The food frequency questionnaire is repeated in the same way for all days of the week.
