# Supplementary material for: Nutritional Biomarkers for the Prediction of Response to Anti-TNF-α Therapy in Crohn’s Disease: New Tools for New Approaches
Source: Nutrients. 2024 Jan 17;16(2):280. doi: 10.3390/nu16020280 (PMC10818399; doi:10.3390/nu16020280)
Supplement: Supplementary file 1 [file nutrients-16-00280-s001.zip › Supplementary File S2 figures amended.pdf]

nutrients

MDPI

Supplementary Figures

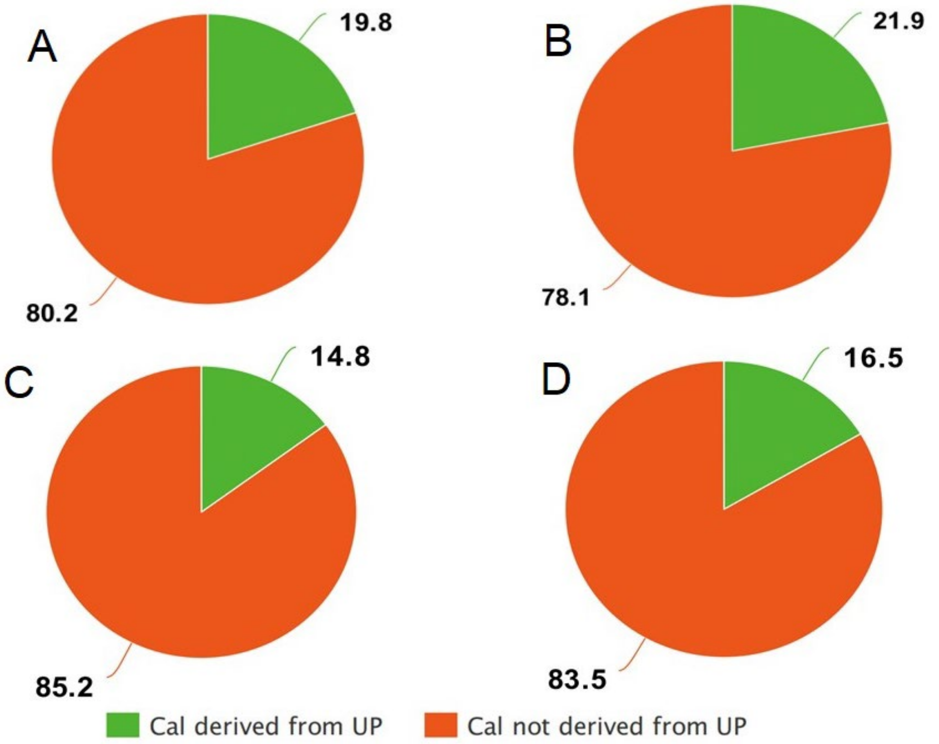

Figure S1. Percentage of calories derived or not from ultra-processed (UP) foods in CD-A at V3 (**A**), CD-R at V3 (**B**), CD-A at V1 (**C**), and CD-R at V1 (**D**).

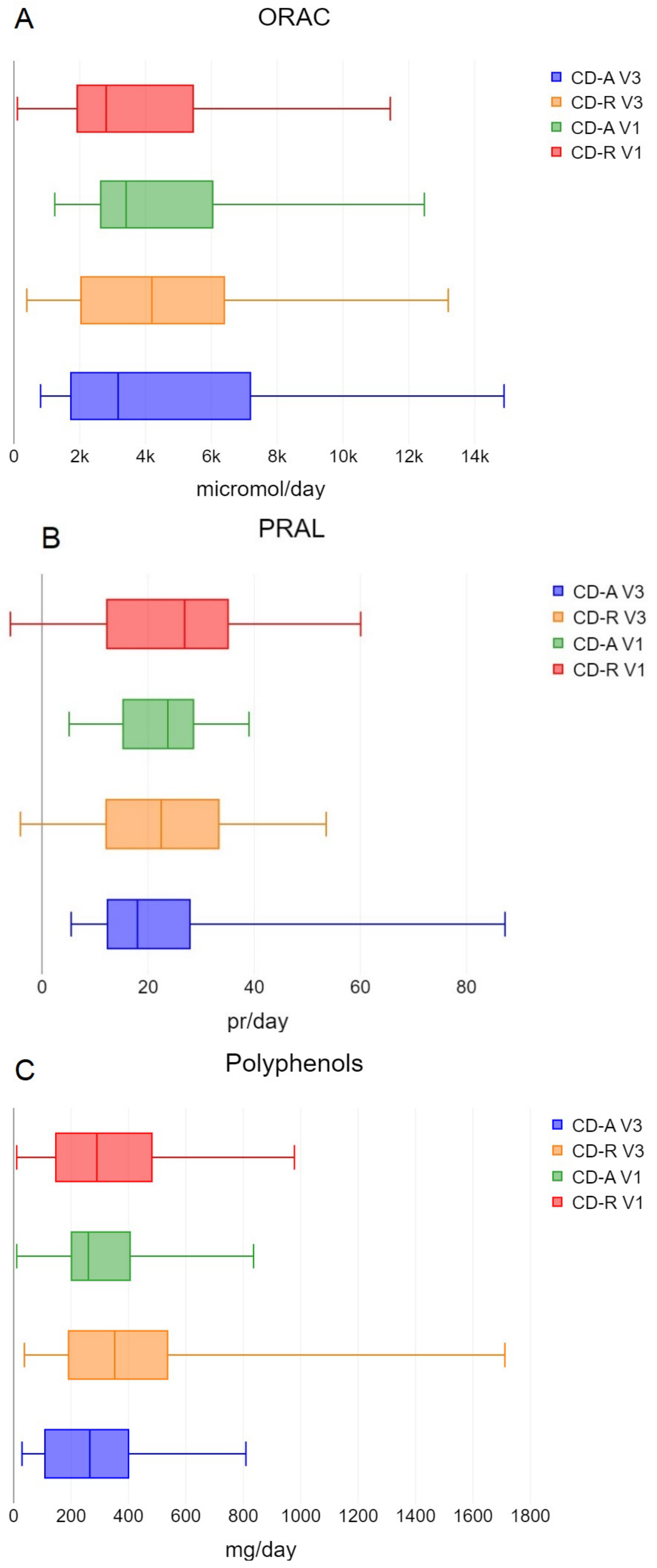

Figure S2. ORAC (**A**), PRAL (**B**), and total Polyphenols (**C**) intake.
